# Attitude, perception, and experience of simulation-based medical learning: A cross-sectional study of respiratory therapy students in Saudi Arabia

**DOI:** 10.1097/MD.0000000000045130

**Published:** 2025-10-10

**Authors:** Abdulelah M. Aldhahir, Mohammed M. Alyami, Raghad A. Alshehri, Ruyuf A. Alnashibi, Ahmed H. Alasimi, Ali S. AlQahtani, Musaad J. Alghamdi, Abdullah A. Alqarni, Jaber S. Alqahtani, Abdallah Y. Naser, Hassan Alwafi, Saeed M. Alghamdi, Rayan A. Siraj

**Affiliations:** a Respiratory Therapy Program, Department of Nursing, College of Nursing and Health Sciences, Jazan University, Jazan, Saudi Arabia; b Health Research Center, Jazan University, Jazan, Saudi Arabia; c Respiratory Therapy Department, Batterjee Medical College, Khamis Mushait, Saudi Arabia; d National Heart and Lung Institute, Imperial College London, London, UK; e Department of Respiratory Therapy, Georgia State University, Atlanta, GA; f Respiratory Therapy Department, King Saud Bin Abdulaziz University for Health Sciences, Jeddah, Saudi Arabia; g Department of Respiratory Therapy, Faculty of Medical Rehabilitation Sciences, King Abdulaziz University, Jeddah, Saudi Arabia; h Respiratory Therapy Unit, King Abdulaziz University Hospital, Jeddah, Saudi Arabia; i Department of Respiratory Care, Prince Sultan Military College of Health Sciences, Dammam, Saudi Arabia; j Department of Applied Pharmaceutical Sciences and Clinical Pharmacy, Faculty of Pharmacy, Isra University, Amman, Jordan; k Department of Pharmacology and Toxicology, College of Medicine, Umm Al-Qura University, Makkah, Saudi Arabia; l Clinical Technology Department, Respiratory Care Program, Faculty of Applied Medical Sciences, Umm Al-Qura University, Makkah, Saudi Arabia; m Department of Respiratory Therapy, College of Applied Medical Sciences, King Faisal University, AL-Ahsa, Saudi Arabia.

**Keywords:** education, respiratory therapy students, Saudi Arabia, simulation-based learning, teaching

## Abstract

Simulation-based education (SBE) is increasingly recognized as an effective pedagogical approach in health sciences, fostering technical and interpersonal skills essential for clinical practice. However, limited research has focused on respiratory therapy (RT) students’ attitudes toward SBE in Saudi Arabia. This study aimed to evaluate RT students’ perceptions, attitudes, and experiences with simulation-based learning across the kingdom. A validated cross-sectional survey, using the KidSIM scale, was conducted to assess attitudes of RT students toward various domains of SBE. Descriptive statistics summarized responses, while *t*-tests and ANOVA analyzed differences between demographic and academic groups. Multiple linear regression identified key predictors of positive attitudes. A total of 1290 RT students completed the survey, with 64.2% (n = 828) female and 31.1% (n = 401) in their fourth academic year. Over half of the participants (56.9%, n = 734) were enrolled in private universities. Most students reported prior experience with interprofessional education (IPE) simulation activities (83.5%), and 43.5% had a grade point average (GPA) between 3.50 and 4.49. Overall, students demonstrated positive attitudes toward SBE, with a mean KidSIM score of 118 ± 31 out of 150. Multiple linear regression identified gender (β = 3.64, 95% CI: 0.36–6.92, *P* = .030), academic year (β = 1.65, 95% CI: 0.02–3.29, *P* = .047), GPA (β = 9.23, 95% CI: 7.29–11.18, *P* < .001), university sector (β = 9.64, 95% CI: 6.37–12.91, *P* < .001), and critical care experience (β = 2.65, 95% CI: 1.70–3.60, *P* < .001) as significant predictors of positive attitudes toward simulation. RT students in Saudi Arabia generally have a positive attitude toward SBE, recognizing it as an effective learning tool, especially for enhancing communication and collaborative skills essential for clinical practice. Progressive integration of simulation throughout RT curricula is recommended to optimize educational outcomes. Future longitudinal studies are needed to explore the long-term impact of SBE on clinical competence and patient care outcomes.

## 1. Introduction

Simulation-based education (SBE) has emerged as a cornerstone of contemporary healthcare training, offering immersive, experiential learning that bridges the gap between theoretical knowledge and real-world clinical practice.^[1]^ By replicating complex, high-stakes scenarios in a safe and controlled environment, SBE enables students to develop essential clinical skills, enhance critical thinking, and make informed decisions without jeopardizing patient safety.^[2,3]^ This pedagogical approach is particularly valuable in dynamic and culturally evolving healthcare systems, such as that of Saudi Arabia, where there is a national drive under the Vision 2030 framework to elevate the quality and accessibility of healthcare services.^[4]^ Vision 2030 emphasizes modernizing education and developing a skilled healthcare workforce, and integrating SBE into health curricula directly supports these goals by promoting competency-based training and aligning graduates’ skills with national healthcare priorities.^[5]^

Unlike traditional didactic methods, SBE promotes a learner-centered approach that integrates cognitive, psychomotor, emotional, and social learning domains.^[6,7]^ This holistic model has been shown to improve learner engagement, foster reflective practice, and strengthen clinical reasoning and knowledge retention.^[8]^ Moreover, participation in simulation-based training is associated with increased confidence, enhanced competence, and the development of a strong professional identity among healthcare students.^[9,10]^ As such, educators and policymakers increasingly advocate for the integration of SBE into health curricula.^[11]^

A growing body of international literature supports the effectiveness of SBE across diverse disciplines. In Canada, for example, nursing and respiratory care students reported improved preparedness and decision-making following high-fidelity simulation training.^[12,13]^ Similarly, studies from the United Kingdom highlight how allied health students value simulation as a practical tool for translating theoretical concepts into action, especially in managing high-risk clinical scenarios.^[14]^ Within Saudi Arabia, recent surveys among nursing and health sciences students have revealed overwhelmingly positive attitudes toward simulation. These attitudes were more favorable among senior students and those with higher academic performance, suggesting a strong correlation between clinical maturity and appreciation of simulation.^[15–17]^

In response to global trends and national educational goals, medical and allied health programs across Saudi Arabia have increasingly adopted simulation-based modules to align with international standards and improve clinical competency.^[18,19]^ The saudi commission for health specialties has also underscored the importance of simulation in training and licensure pathways.^[18]^ However, the success of simulation programs depends not only on curricular design but also on students’ attitudes, perceptions, and engagement.

Given the growing role of simulation in healthcare education and the limited research focusing specifically on respiratory therapy (RT) students in Saudi Arabia, this study aims to evaluate RT students’ attitudes, perceptions, and experiences with SBE. Understanding their views is critical to optimizing simulation practices and ensuring the alignment of educational strategies with learners’ needs and expectations. Based on prior literature, we hypothesized that RT students would demonstrate overall positive attitudes toward SBE and perceive it as an effective and safe learning approach that bridges theoretical knowledge with practical application and supports their readiness for real-world clinical practice.

## 2. Materials and methods

### 2.1. Study design and study population

A descriptive cross-sectional survey targeting RT students across Saudi Arabia was conducted over a two-month period. Data collection was carried out between January 21 and March 23, 2024, using an online questionnaire disseminated to several RT programs and through various social media platforms.

### 2.2. Sampling strategy

A convenience sampling strategy was adopted to recruit RT students across Saudi Arabia. The inclusion criteria were clearly stated in the opening section of the online survey, which specified eligibility for students enrolled from the second academic year up to the internship year. First-year pre-medical students were excluded, as they had not yet entered the core RT curriculum. The survey invitation was circulated through various social media platforms and was sent to several RT programs to facilitate broad outreach. Prior to accessing the main questionnaire, participants were required to confirm their consent by answering a compulsory question regarding their willingness to participate. To maintain data quality, the survey system was configured to accept only one response per participant, thereby minimizing the risk of duplicate entries.

### 2.3. Study instrument

A structured and validated questionnaire was employed to evaluate RT students’ attitudes, perceptions, and experiences related to educational simulation in Saudi Arabia. The survey was composed of 2 main sections and included a total of 38 items.

The first section consisted of 8 questions designed to collect demographic and academic information. These included participants’ gender, age, academic year, cumulative grade point average (GPA), university sector, prior participation in interprofessional education (IPE) simulation, previous involvement in team-based learning, and level of clinical exposure to critical care environments.

The second section utilized the KidSIM attitudes toward SBE scale.^[15]^ This validated instrument comprises 30 items that assess students’ attitudes and perceptions toward simulation-based learning, particularly within IPE contexts. Each item is rated on a 5-point Likert scale, ranging from 1 (“strongly disagree”) to 5 (“strongly agree”), yielding a possible total score between 30 and 150. Higher scores indicate more favorable attitudes toward SBE. The scale does not include reverse-coded items and covers 5 core domains: the relevance of simulation, opportunities for IPE, communication, professional roles and responsibilities, and situational awareness. The full survey required approximately 10 minutes to complete. All items were mandatory to ensure comprehensive data collection, and technical safeguards were applied to prevent multiple submissions from the same participant.

Regarding local validation, we conducted pilot testing of the KidSIM scale among 15 RT students, which confirmed the clarity, relevance, and cultural appropriateness of the items for the Saudi context. Reliability analysis in our study sample showed excellent internal consistency, with Cronbach’s alpha coefficients as follows: Relevance of simulation, 0.940; Opportunities for IPE, 0.943; Communication, 0.942; Roles and responsibilities, 0.934; Situational awareness, 0.816; and an overall Cronbach alpha of 0.983. These findings support both the reliability and the local validity of the scale in this population.

### 2.4. Ethical consideration

Prior to initiating data collection, ethical approval was obtained from the Research Ethics Committee at King Abdulaziz University (Approval Date: November 20, 2023; Reference No. FMRS-EC2024-015). The study methods were conducted in accordance with the principles of the Declaration of Helsinki.

### 2.5. Sample size

Based on the most recent estimate of RT students in Saudi Arabia (N = 1297 in 2022)^[20]^ and anticipating an increase to about 2000 students due to newly established programs, the required sample size ranged from approximately 323 (margin of error 5%) to 870 (margin of error 2.5%). In this study, 1290 RT students were recruited in 2024, exceeding the required number and ensuring adequate statistical power and precision.

### 2.6. Statistical analysis

Statistical analyses were conducted using SPSS version 28 (IBM Corp., Armonk), following data collection through an online survey and initial organization in Microsoft Excel. Descriptive statistics, including means (M), standard deviations, and percentages (%), were used to summarize students’ attitudes and perceptions toward educational simulation. Independent sample *t*-tests and one-way ANOVA were applied to examine differences across groups based on demographic and academic variables. To identify significant predictors of positive attitudes, a multiple linear regression analysis was performed, with the total attitude score treated as the dependent variable and all student characteristics entered as independent variables. A *P*-value of <.05 was considered statistically significant.

## 3. Results

### 3.1. Demographic data of the study participants

Overall, 1290 RT students across 16 different universities responded to the online survey. The mean (±SD) age of RT students was 23 (±4) years with a higher proportion of female students (828, or 64.2%). The highest proportion of RT students reported a cumulative GPA between 3.50 and 4.49 (561, or 43.5%) followed by a cumulative GPA between 4.50 and 5.00 (459, or 34.9%). More than half of RT students studied at a private university (734, or 56.9%) and 1077 (83.5%) had previously participated in IPE simulation (Table 1).

**Table 1:** Demographic data of the study participants (N = 1290).

Variables	Frequency (%), or mean (±SD)
Gender *n* (%)	
Male	462 (35.8%)
Female	828 (64.2%)
Age mean (±SD)	23 ± 4
Academic year *n* (%)	
Second year	342 (26.5%)
Third year	380 (29.5%)
Fourth year	401 (31.1%)
Internship	167 (12.9%)
cGPA, *n* (%)	
<2.50	68 (5.3%)
2.50–3.49	211 (16.4%)
3.50–4.49	561 (43.5%)
4.50–5.00	450 (34.9%)
University sector *n* (%)	
Governmental university	556 (43.1%)
Private university	734 (56.9%)
Previous participation in inter-professional education (IPE) simulation *n* (%)	
Yes	1077 (83.5%)
No	213 (16.5%)
Previous team based learning, *n* (%)	
Workshop	270 (20.9%)
Seminar	171 (13.3%)
Course	631 (48.9%)
Work experience	218 (16.9%)
Critical care experience, n (%)	
None	210 (16.3%)
<1 wk	139 (10.8%)
<2 wk	207 (16%)
<3 wk	267 (20.7%)
1 mo	182 (14.1%)
>1 mo	285 (22.1%)

Data are presented as frequency and percentage or mean ± standard deviation.

GPA = grade point average.

RT students were also asked about previous simulated team-based learning and critical care experience. Almost half of RT students acquired simulated team-based learning from their course (631, 48.9%). Regarding critical care experience, 285 (22.1%) reported critical care experience of more than a month followed by 267 (20.7%) who reported having less than 3 weeks of critical care experience (Table 1).

### 3.2. Students attitudes and perceptions toward interprofessional education simulation

RT students’ attitudes and perception toward IPE simulation was encouraging with a total mean score of 118 (±31) on the KidSIM scale, which ranges between 30 and 150. Out of 1290 RT students, only 121 (9%) reported a score below the KidSIM scale average. The full details of mean (±) of total score and each item score of attitude and perception toward IPE simulation using KidSIM scale are presented in Table 2.

**Table 2:** Students’ attitudes and perceptions toward interprofessional education simulation (N = 1290).

Item	Mean (±SD)
Relevance of simulation	
Simulation is a good environment for learning with other health care professionals	3.79 (±1.31)
Simulation supports opportunities to change attitudes	3.83 (±1.28)
Opportunities to practice teamwork can help students learn about inter-professional roles	3.89 (±1.26)
Opportunities to learn with other health care professionals has increased my understanding of their roles	3.95 (±1.26)
Simulation is a good tool for practicing team decision-making skills	3.93 (±1.24)
Deliberate practice can improve clinical decision-making skills	3.93 (±1.25)
Opportunities for interprofessional education (IPE)	
Learning with other professionals is important to collaboration	3.85 (±1.30)
Opportunities to learn with other professionals should be a priority in my education	3.92 (±1.26)
I want more opportunities to learn with other professionals	3.92 (±1.24)
Shared learning with other team members will improve my ability to understand clinical problems	3.97 (±1.23)
Attitudes about teamwork can change through opportunities to work with other professionals in simulation	3.92 (±1.25)
Learning with other health care professionals before qualification is important for the development of future interprofessional relationships	3.95 (±1.24)
Interprofessional opportunities for learning will improve patient outcomes	3.99 (±1.20)
Communication	
All students should learn how to work in the context of health care teams	3.97 (±1.26)
Team leaders should provide frequent patient updates to other team members	4.01 (±1.23)
Team leaders should encourage team members to ask questions	4.05 (±1.19)
Communication within the team is as important as technical skills	3.99 (±1.22)
Team members providing immediate patient care management should verbalize their activities aloud	3.80 (±1.32)
Team members should paraphrase or repeat back instructions to clarify their understanding	3.95 (±1.23)
Communication in teamwork is important to patient safety	4.03 (±1.21)
The roles of non-leading members of the team are just as important for good team functioning as the role of the leader	3.94 (±1.24)
Roles and responsibilities	
Teamwork practice will provide me with feedback to enhance my ability to provide optimal patient care	3.84 (±1.29)
Monitoring what each team member is doing is important to optimize patient safety	3.90 (±1.30)
Will enhance other team members understanding of my role in patient health care	3.97 (±1.24)
Teamwork practice will help me recognize how best to help other team members complete their tasks	3.94 (±1.22)
It is important for team members to ask for assistance if they need support in completing a task	3.94 (±1.24)
Teamwork practice allows for flexibility in roles during times of crisis	4.02 (±1.19)
Situation awareness	
I will speak up if I perceive a problem regardless of who might be affected	3.63 (±1.43)
Patient care is improved when all team members have a shared understanding of the assessment and treatment	3.98 (±1.22)
Team leaders should provide frequent summaries of patient findings to keep team members oriented to patient needs	3.99 (±1.21)
Total score (30–150)	118 ± 31

Data are presented as mean ± standard deviation.

The mean (±SD) of KidSIM 5 domains (Relevance of Simulation, Opportunities for IPE, Communication, Roles and Responsibilities, and Situation Awareness) were assessed and presented in Table 3. The communication subscale mean (±SD) was 31.73 (±8.37), followed by Opportunities for IPE with mean (±SD) of 27.53 (±7.53), Roles and responsibilities 23.62 (±6.49), Relevance of simulation 23.32 (±6.68), and Situation awareness 11.60 (±3.31) (Table 3).

**Table 3:** The total mean scores on the 5 domains of the KidSIM scale.

Domian	Number of items	Possible range	Mean (±SD)
Relevance of simulation	6	6–30	23.32 (±6.68)
Opportunities for interprofessional education (IPE)	7	7–35	27.53 (±7.53)
Communication	8	8–40	31.73 (±8.37)
Roles and responsibilities	6	6–30	23.62 (±6.49)
Situation awareness	3	3–15	11.60 (±3.31)

Data are presented as mean ± standard deviation.

### 3.3. RT student characteristics that influence attitudes and perceptions toward simulation

ANOVA test was conducted to examine the differences between means total score, which represent students’ attitudes and perceptions toward IPE simulation and students’ characteristics including gender, academic year, cumulative GPA, university sector, previous participation in IPE simulation, previous team-based learning, and duration of critical care experiences.

**Figure 1.**
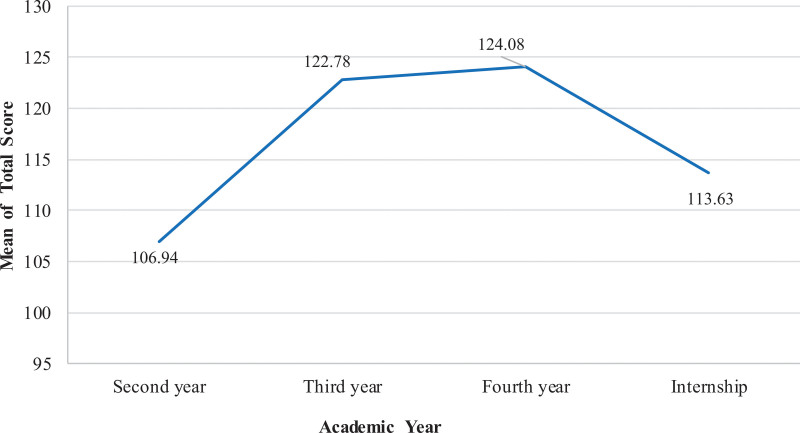
Mean KidSIM scale scores of RT students based on academic year. Fourth-year students had the highest mean scores, while second-year students had the lowest. RT = respiratory therapy.

The ANOVA results showed no significant differences were noted between male and female RT students mean total score (Male: 115.64 ± 30 vs Female: 119 ± 30; *P* = .059). Moreover, significant differences were reported in mean total score and academic year with highest mean score reported for fourth-year RT students (Second year: 106.94 ± 33; Third year: 122.78 ± 26; Fourth year: 124 ± 29; Internship: 133.63 ± 32; *F* = 25.7; *P* < .001) (Fig. 1).

**Figure 2.**
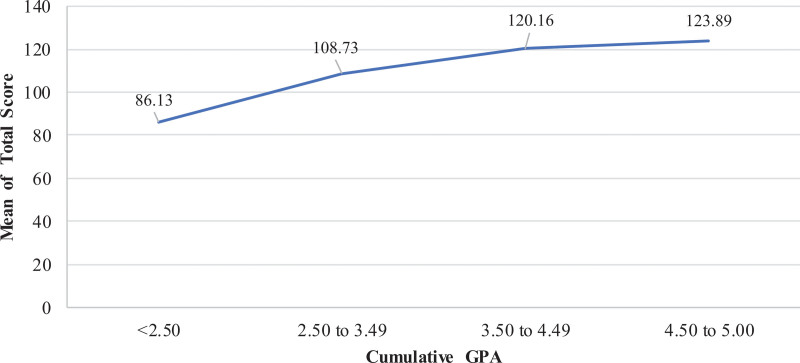
Mean KidSIM scale scores of RT students based on cumulative GPA. Students with a cGPA of 4.50–5.00 had the highest mean scores, whereas those with a cGPA below 2.50 had the lowest mean scores. GPA = grade point average, RT = respiratory therapy.

The ANOVA results showed significant differences in mean total score of KidSIM scale for RT students with different cumulative GPA with highest mean score for RT students with cGPA between 4.50 and 5.00 (123.89 ± 29), followed by cGPA between 3.50 and 4.49 (120.16 ± 28), cGPA between 2.50 and 3.49 (108.73 ± 31), and cGPA < 2.50 (86.13 ± 34; *F* = 40.866; *P* < .001) (Fig. 2).

**Figure 3.**
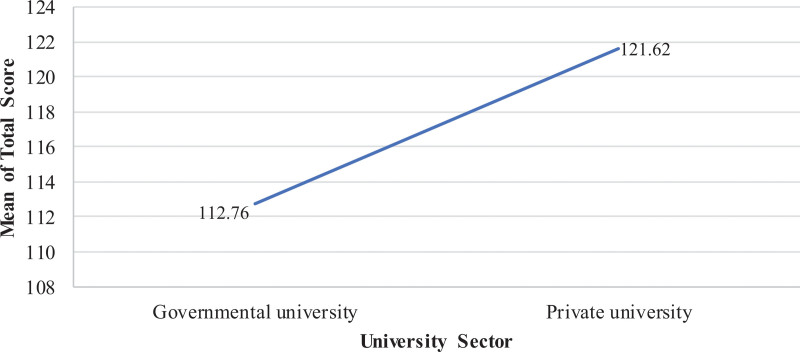
Mean KidSIM scale scores of RT students based on university sector. Students enrolled in a private university demonstrated higher mean scores compared to those in a government university. RT = respiratory therapy.

Additionally, significant differences in total mean scores were reported between RT students in the government university compared to private university with a higher mean score in KidSIM scale for RT students in private university (Private university: 121.62 ± 32 vs Government university: 112.76 ± 28; *F* = 26.989; *P* < .001) (Fig. 3).

**Figure 4.**
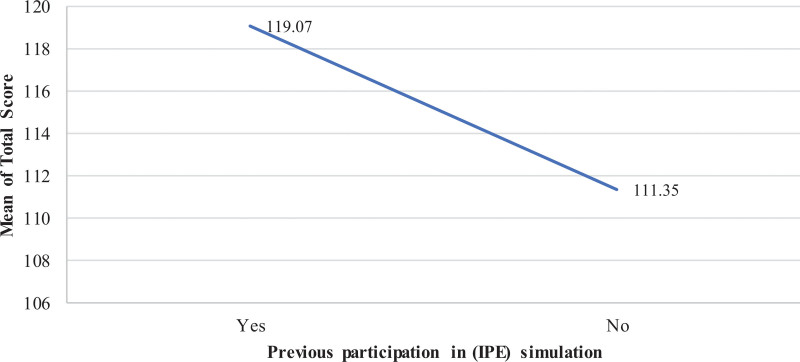
Mean KidSIM scale scores of RT students based on previous participation in IPE simulation. Students with prior IPE simulation experience demonstrated higher mean scores compared to those without such experience. IPE = interprofessional education, RT = respiratory therapy.

Previous participation in IPE simulation was also examined and demonstrated that RT students with previous participation in IPE simulation had significantly higher total mean score in the KidSIM scale (119.07 ± 31) compared to RT students with on prior participation (111.35 ± 27.59; *F* = 11.378; *P* < .001) (Fig. 4).

**Figure 5.**
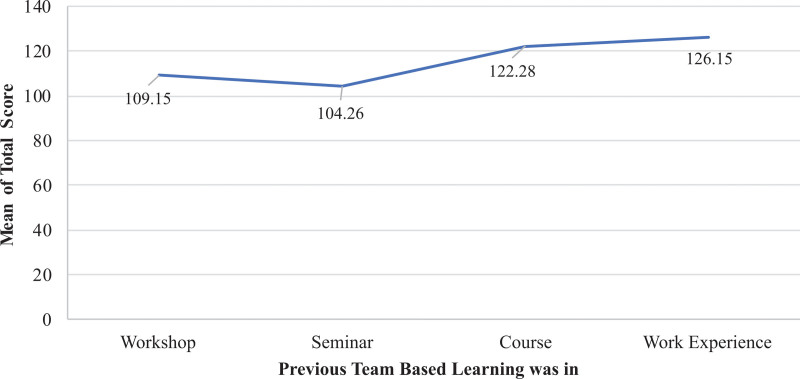
Mean KidSIM scale scores of RT students based on previous team-based learning experience. Students with work experience reported the highest mean score, while those with prior seminar experience had the lowest. RT = respiratory therapy.

Type of teamwork experience was also assessed and revealed that RT students who gained their previous team-based learning from work experience reported the highest mean score in the KidSIM scale followed by course, workshop, and seminar (work experience: 126.15 ± 29; course: 122.28 ± 24; workshop: 109.15 ± 39; seminar: 104.26 ± 30; *F* = 30.086; *P* < .001) (Fig. 5).

**Figure 6.**
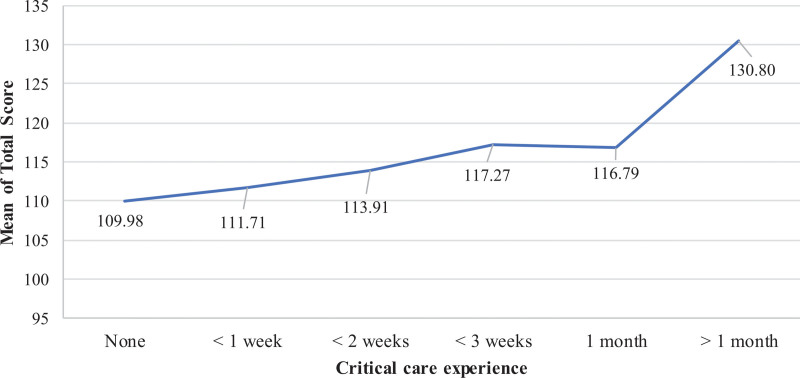
Mean total KidSIM scores of RT students based on the duration of critical care experience. Students with longer critical care exposure (>1 month) demonstrated higher mean scores compared to those with shorter or no experience. RT = respiratory therapy.

With regards to duration of critical care experiences, the highest reported total mean score of KidSIM scale was reported for RT students with more than a month of critical care experience (130.49 ± 26), followed by RT students with less than 3 weeks of critical experience (117.27 ± 25), RT students with a month of critical care experience (116.79 ± 25), RT students with less than 2 weeks of critical care experience (113.91 ± 30), RT students with less than a week of critical care experience (111.71 ± 35), and RT students with no none critical care experience (109.98 ± 39; *F* = 15.125; *P* < .001) (Fig. 6).

ANOVA test was conducted to examine differences in subscale scores, including Relevance of simulation, Opportunities for IPE, Communication, Roles and responsibilities, and Situation awareness with students’ characteristics including gender, academic year, cumulative GPA, university sector. There were significant differences only between male and female RT students with regard to Relevance of simulation (Male: 22.54 ± 6.8 vs Female: 23.75 ± 6.5; *F* = 9.775; *P* = .002) and Communication (Male: 31.10 ± 8.25 vs Female: 32.08 ± 8.42; *F* = 4; *P* = .045). Additionally, there were significant differences between different RT students’ academic year with regard to Relevance of simulation (Second year: 20.94 ± 7; Third year: 24.24 ± 6; Fourth year: 24.69 ± 6; Internship: 22.79 ± 6; *F* = 24.166; *P* < .001), Opportunities for IPE (Second year: 25.06 ± 8; Third year: 28.68 ± 6; Fourth year: 28.94 ± 7; Internship: 26.56 ± 8; *F* = 21,821; *P* < .001), Communication (Second year: 28.75 ± 9; Third year: 33.17 ± 7; Fourth year: 33.37 ± 8; Internship: 30.61 ± 9; *F* = 25.729; *P* < .001), Roles and responsibilities (Second year: 21.54 ± 7; Third year: 24.71 ± 5; Fourth year: 24.85 ± 6; Internship: 22.43 ± 7; *F* = 23.067; *P* < .001), and Situation awareness (Second year: 10.64 ± 3; Third year: 11.97 ± 3; Fourth year: 12.23 ± 3; Internship: 11.24 ± 3; *F* = 17.324; *P* < .001). Similarly, there were significant differences between RT students’ with different cGPA and Relevance of Simulation (cGPA < 2.50: 16.47 ± 7; cGPA 2.50–3.49: 21.36 ± 7; cGPA 3.50–4.49: 23.79 ± 6; cGPA 4.50–5.00: 24.68 ± 6; *F* = 40.513; *P* < .001), Opportunities for IPE (cGPA < 2.50: 20.06 ± 9; cGPA 2.50–3.49: 25.27 ± 8; cGPA 3.50–4.49: 28.10 ± 7; cGPA 4.50–5.00: 29.00 ± 7; *F* = 38.611; *P* < .001), Communication (cGPA < 2.50: 23.32 ± 10; cGPA 2.50–3.49: 29.26 ± 8; cGPA 3.50–4.49: 32.32 ± 8; cGPA 4.50–5.00: 33.42 ± 8; *F* = 39/285; *P* < .001), Roles and responsibilities (cGPA < 2.50: 17.54 ± 7; cGPA 2.50–3.49: 21.97 ± 6; cGPA 3.50–4.49: 24.10 ± 6; cGPA 4.50–5.00: 24.71 ± 6; *F* = 31.813; *P* < .001), and Situation Awareness (cGPA < 2.50: 8.74 ± 4; cGPA 2.50–3.49: 10.88 ± 3; cGPA 3.50–4.49: 11.85 ± 3; cGPA 4.50–5.00: 12.07 ± 3; *F* = 25.884; *P* < .001). Finally, there were significant differences between RT students’ who studied at governmental or private university and Relevance of simulation (Governmental university: 22.20 ± 6; Private university: 24.17 ± 7; *F* = 28.213; *P* < .001), Opportunities for IPE (Governmental university: 26.32 ± 7; Private university: 28.44 ± 8; *F* = 25.463; *P* < .001), Communication (Governmental university: 30.46 ± 8; Private university: 32.69 ± 9; *F* = 22.729; *P* < .001), Roles and responsibilities (Governmental university: 22.63 ± 6; Private university: 24.37 ± 6; *F* = 23.251; *P* < .001), and Situation Awareness (Governmental university: 11.15 ± 3; Private university: 11.95 ± 3; *F* = 18.994; *P* < .001).

### 3.4. Predictors of positive attitudes and perception toward simulation

A multiple linear regression was conducted to examine predictors of students’ attitudes and perceptions toward simulation. The model was statistically significant, *F* (6, 1283) = 33.93, *P* < .001, and explained 13.3% of the variance in the outcome (Adjusted *R*^2^ = 0.133). Several factors were found to be significant predictors. Female students reported higher scores compared to males (*B* = 3.64, *P* = .030), and each increase in academic year was associated with a 1.65-point increase (*P* = .047). GPA had the strongest positive effect (*B* = 9.23, *P* < .001), indicating that higher academic achievement correlates with more favorable simulation attitudes. Students from private universities also scored significantly higher than those from government institutions (*B* = 9.64, *P* < .001). Participation in IPE simulation showed a marginal association (*P* = .057), while critical care experience was a significant predictor (*B* = 2.65, *P* < .001), suggesting that clinical exposure enhances simulation attitudes (Table 4).

**Table 4:** Predictors of positive students’ attitudes and perception toward simulation.

Variable	Beta (95% CI)	SEM	*P*-value
Constant	50.94 (40.40–61.49)	5.38	<.001
Gender	3.64 (0.36–6.92)	1.67	.030
Academic year	1.65 (0.02–3.29)	0.83	.047
GPA	9.23 (7.29–11.18)	0.99	<.001
University sector	9.64 (6.37–12.91)	1.67	<.001
IPE participation	4.38 (−0.13–8.89)	2.30	.057
Critical care experience	2.65 (1.70–3.60)	0.49	<.001

GPA = grade point average, IPE = interprofessional education.

## 4. Discussion

This study provides an important contribution to the growing body of literature on SBE by specifically examining the attitudes, perceptions, and experiences of RT students in Saudi Arabia. Although several studies have previously explored the role of SBE in health sciences education within the region, most have predominantly focused on nursing and medical students,^[16,17,21]^ with limited attention given to the RT discipline.^[15]^ Our findings suggest that overall attitudes toward SBE among RT students tended to be favorable. The mean total KidSIM score (118 ± 31) indicates that students generally perceived simulation as a valuable and effective learning tool. This positive perception appeared to be consistent across different academic years and institutions, indicating widespread acceptance of simulation as an integral component of RT education

In line with broader educational trends, SBE is increasingly recognized as a valuable instructional approach across various health disciplines due to its ability to replicate real-world clinical scenarios and foster active learning.^[22]^ In the present study, students rated the communication and IPE domains most favorably, highlighting the perceived value of simulation in enhancing collaboration, teamwork, and patient-centered communication. These findings are consistent with previous studies reporting that health sciences students particularly appreciated the role of simulation in improving interprofessional interactions, self-confidence, and communication skills.^[23,24]^ The prominence of these domains in student evaluations supports the notion that simulation not only enhances technical proficiency but also plays a pivotal role in developing interpersonal and professional competencies.^[25]^ This dual emphasis on clinical and relational skill-building reflects trends in health professions education, which increasingly recognize the importance of preparing students for complex, real-world interactions with patients and healthcare teams.

Interestingly, GPA emerged as the most significant predictor of positive attitudes toward SBE. This finding aligns with previous research suggesting that health science students with higher academic performance consistently exhibit more favorable perceptions of simulation’s structure, relevance, and educational value.^[15]^ Similarly, a recent survey among nursing students revealed that those with higher GPAs were significantly more appreciative of the benefits of simulation, which has been interpreted as related to their increased motivation and deeper cognitive engagement with complex clinical scenarios.^[16]^ This association may reflect that academically high-performing students often possess characteristics such as intrinsic motivation, self-directed learning habits, and cognitive flexibility traits that enable them to engage more deeply with simulation-based content and derive greater educational benefit.^[26,27]^ These findings highlight the potential importance of designing simulation curricula that are responsive to students’ varying academic capabilities, thereby ensuring that all learners, regardless of academic standing, can fully benefit from the pedagogical advantages of SBE.

In this study, students enrolled in private universities reported significantly higher KidSIM scores than their counterparts from public institutions. One plausible explanation for this disparity could be that students at private universities often have higher monthly expenditures, reflecting greater financial investment in their education. This financial advantage may contribute to improved access to advanced educational resources, including high-quality simulation technologies and better-equipped training facilities.^[28]^ Additionally, comparative evaluations of educational quality have shown that private institutions consistently score higher in their adoption of virtual learning tools, which could enhance the implementation of student-centered teaching methodologies.^[29]^ Such environments might be more conducive to delivering effective SBE, which is essential for the practical development of health science students. Conversely, public universities frequently encounter structural challenges, such as limited budgets and resource constraints, which may restrict their capacity to invest in and expand simulation infrastructure.^[30]^ Targeted investments in simulation infrastructure, structured faculty development, and curriculum-wide integration of SBE could help reduce disparities and support high-quality training.

Furthermore, academic year was identified as a key factor shaping students’ attitudes toward SBE, with fourth-year students exhibiting notably more positive perceptions than their junior peers. This progressive enhancement in attitude might be related to increased exposure to clinical content, deeper familiarity with simulation environments, and more frequent opportunities to apply theoretical knowledge in practical, hands-on contexts. Consistent with these findings, prior studies have reported that students in advanced academic stages tend to express greater satisfaction with simulation technologies compared to those in earlier years.^[16,31]^ Additionally, research indicates that senior students often demonstrate higher levels of self-efficacy and a stronger sense of preparedness for clinical practice following engagement with simulation-based training.^[32]^ These outcomes suggest the critical importance of longitudinally integrating simulation into health sciences curricula may contribute to fostering competence and confidence.

In addition to academic standing, our findings revealed that RT students who had prior exposure to IPE simulations, particularly when combined with previous team-based learning experiences, achieved the highest mean scores on the KidSIM scale. This aligns with existing literature, which emphasizes that early and repeated engagement in IPE activities can enhances collaborative competencies such as communication, teamwork, and problem-solving, all of which are fundamental to ensuring patient safety and delivering high-quality care.^[33–35]^ Through structured, realistic scenarios, IPE simulations provide opportunities for RT students to develop interprofessional skills within a safe environment. These experiences allow students to benefit from diverse perspectives across allied health disciplines, reinforcing the importance of shared learning and mutual respect.^[36]^ Moreover, practical exposure to interprofessional collaboration may prepare students for complex clinical realities, supporting the growing body of evidence that such competencies are directly linked to improved healthcare outcomes.^[37]^ This synergistic integration of theoretical instruction and hands-on application highlights the indispensable role of IPE in cultivating well-rounded, practice-ready healthcare professionals capable of navigating the intricacies of contemporary healthcare delivery.^[38,39]^

With regard to gender-based differences, our analysis revealed that female students demonstrated significantly more positive attitudes toward SBE than their male counterparts. This finding aligns with the work of Eltaib et al, who reported that a majority of female nursing students held favorable perceptions of simulation, suggesting that gender may influence attitudes toward educational tools.^[16]^ Similarly, Karataş et al observed that female students in special and physical education programs often expressed more positive views toward inclusive and experiential practices compared to males, further supporting the notion of gender-based differences in educational engagement.^[40]^ Simulations, by offering immersive and structured learning experiences, may resonate more with female learners, enhancing their receptivity and perceived value of such methods.^[41]^ These differences could reflect broader gendered patterns of socialization, wherein females are often encouraged to collaborate, reflect, and engage empathetically in academic contexts traits that align well with the goals and design of simulation-based learning.^[42]^ Collectively, these findings underscore the importance of considering gender-specific learning preferences when designing and implementing simulation-based curricula to ensure optimal educational outcomes for all students.

The findings provide potential implications for RT education in Saudi Arabia. To fully realize the benefits of SBE, it could be systematically integrated into the curriculum from the early stages, with increasing complexity and intensity over time. Equally important is the need to prioritize comprehensive instructor training to ensure that simulation activities are pedagogically effective and aligned with defined learning outcomes. Moreover, institutions, particularly public universities, should be encouraged and supported to invest in high-quality simulation infrastructure and technology to provide consistent, hands-on learning experiences throughout all 4 undergraduate years. Lastly, simulation should not be viewed as a standalone educational tool but rather embedded within the broader clinical training framework to enhance knowledge retention, skill acquisition, and clinical competence.

### 4.1. Strengths and limitations

This study offers several important strengths, as it provides a comprehensive assessment of RT students’ attitudes, perceptions, and experiences with SBE in Saudi Arabia, an area that has received limited prior attention. The inclusion of a well-representative sample from both public and private universities enhances the generalizability of the findings across the country. Additionally, the use of a validated survey instrument (KidSIM scale) strengthens the reliability and comparability of the results. However, certain limitations should be acknowledged. The use of a convenience sampling method, coupled with reliance on recruitment through RT programs and social media platforms, may have introduced selection bias, as students with greater interest in simulation could have been more likely to participate. The use of self-reported survey data may also introduce reporting bias, as responses could be influenced by social desirability. Moreover, the cross-sectional nature of the study limits the ability to infer causal relationships. Although the sample included students from diverse institutional backgrounds, the study did not directly assess heterogeneity in simulation resources or faculty competence, which may also influence student experiences. Future studies should adopt longitudinal or mixed-method approaches and examine institutional-level factors to further enhance the understanding and application of SBE in RT programs.

## 5. Conclusion

This study revealed that RT students in Saudi Arabia generally report positive attitudes toward SBE, particularly in areas related to communication, interprofessional collaboration, and understanding clinical roles. Factors such as higher GPA, advanced academic standing, enrollment in private universities, prior critical care exposure, and previous participation in interprofessional simulation were associated with more favorable perceptions. These findings suggest that simulation may contribute to enhancing students’ readiness for clinical practice and collaborative learning. To further clarify its educational impact, simulation could be progressively incorporated into RT curricula, with future longitudinal studies needed to evaluate its sustained effects on students’ clinical competence, confidence, and patient care outcomes.

## Author contributions

**Conceptualization:** Abdulelah M. Aldhahir.

**Data curation:** Mohammed M. Alyami, Abdullah A. Alqarni, Jaber S. Alqahtani, Raghad A. Alshehri, Ruyuf A. Alnashibi.

**Formal analysis:** Abdulelah M. Aldhahir, Ahmed H. Alasimi.

**Investigation:** Abdallah Y. Naser, Hassan Alwafi.

**Methodology:** Abdulelah M. Aldhahir, Saeed M. Alghamdi, Rayan A. Siraj.

**Project administration:** Abdallah Y. Naser, Hassan Alwafi, Saeed M. Alghamdi.

**Resources:** Mohammed M. Alyami.

**Supervision:** Abdulelah M. Aldhahir, Rayan A. Siraj.

**Validation:** Mohammed M. Alyami, Abdullah A. Alqarni, Jaber S. Alqahtani.

**Writing – original draft:** Abdulelah M. Aldhahir, Raghad A. Alshehri, Ruyuf A. Alnashibi, Ahmed H. Alasimi, Ali S. AlQahtani, Musaad J. Alghamdi.

**Writing – review & editing:** Abdulelah M. Aldhahir, Mohammed M. Alyami, Raghad A. Alshehri, Ruyuf A. Alnashibi, Ahmed H. Alasimi, Ali S. AlQahtani, Musaad J. Alghamdi, Abdullah A. Alqarni, Jaber S. Alqahtani, Abdallah Y. Naser, Hassan Alwafi, Saeed M. Alghamdi, Rayan A. Siraj.
